# Surgical management of a traumatic intralenticular abscess: a case report

**DOI:** 10.11604/pamj.2022.41.81.30914

**Published:** 2022-01-30

**Authors:** Jihene Sayadi, Manel Mekni, Zeineb Kallel, Racem Choura, Dhouha Gouider, Ines Malek, Leila Nacef

**Affiliations:** 1 Hedi Raies Institute of Ophthalmology, Tunis El-Manar University, Tunis, Tunisia

**Keywords:** Cataract extraction, intralenticular abscess, trauma, case report

## Abstract

lntralenticular abscess is a very rare entity that has been described after penetrating trauma, intraocular surgery and metastatic spread. We report a case of intralenticular abscess treated surgically by phacoemulsification with good postoperative results. A 32-year-old patient presented with right eye redness and defective vision of 4 days following thorn injury. The visual acuity was limited to counting fingers. Anterior segment examination revealed healed lamellar corneal tear, 3+ cells in the anterior chamber, iris synechia and heterogeneous opacity of the crystalline lens. Vitreous and fundus were normal. Initially, we prescribed topic and systemic antimicrobial treatment. Lens extraction was performed 1 week later by phacoemulsification with primary intraocular lens implantation. The immediate postoperative recovery was uneventful. The visual acuity at last follow-up was 9/10. In the current case, lens extraction associated with systemic and local antimicrobial treatment allowed infection control and good visual outcome.

## Introduction

Intralenticular abscess is a rare condition described following penetrating trauma, intraocular surgery or infection metastatic spread [1,2]. The clinical features, predisposing factors and appropriate treatment are not well codified. Actually, only few cases have been reported in the literature. Herein, we report a case of a crystalline nuclear abscess that occurred in a patient with a corneal thorn injury successfully managed by early surgical approach.

## Patient and observation

**Patient information:** a 32-year-old man with no medical or surgical history presented with a chief complaint of red painful right eye. A history taking revealed a traumatic thorn accident four days before the symptomatology onset.

**Figure 1:**
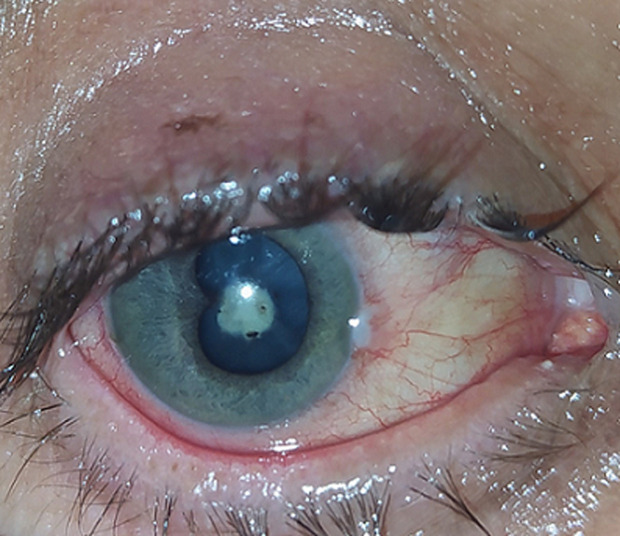
baseline photography of the right eye showing temporal iris synechia and central lens abscess

**Clinical findings:** at presentation, best corrected visual acuity in the right eye was limited to counting fingers. The slit lamp examination showed no thorn in conjunctiva nor cornea. Further examination revealed an important conjunctival injection, a perikeratic circle, a healed corneal tear, 3+ cells in the anterior chamber and a temporal iris synechia. In addition, a 3x4mm well demarcated circular and centered cheesy white opacity in the crystalline lens was present. No perilenticular exsudative reaction nor vitreous inflammation were noted. Fundus examination was unremarkable (Figure 1). The left eye evaluation was within normal limits.

**Figure 2:**
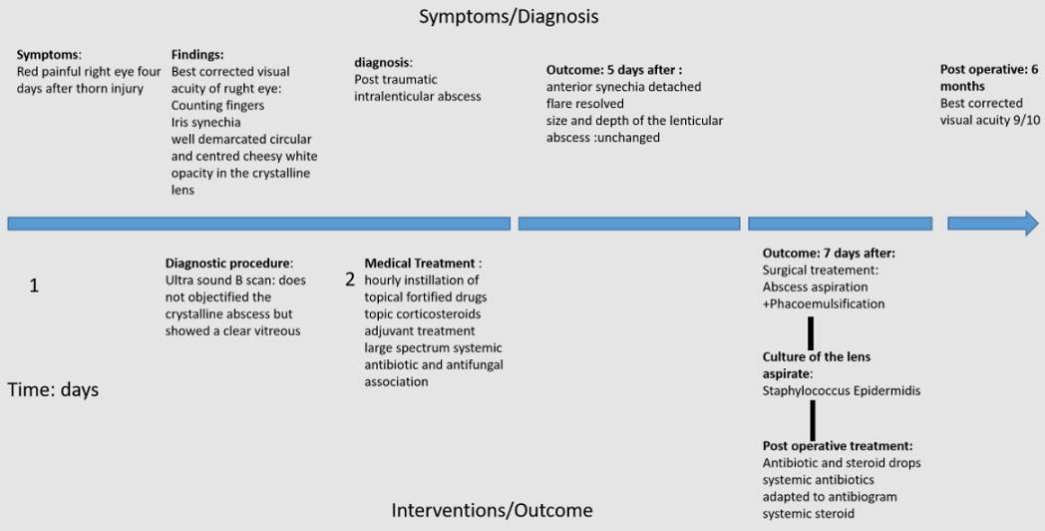
timeline of current episodes from presentation to patient's management

**Timeline of current episode:** the key steps from presentation to patient´s management are summarized in Figure 2.

**Figure 3:**
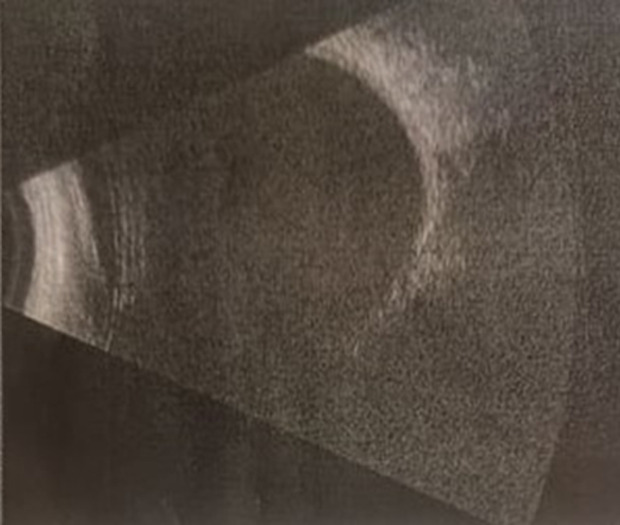
ultrasound B scan of the right eye showing a clear vitreous and no intraocular foreign body

**Diagnostic assessment:** the ultrasound B scan has not shown the crystalline abscess but showed a clear vitreous (Figure 3). Neither optical coherence tomography (OCT) of anterior segment nor ultra-bio microscopy (UBM) could be realized because of limited technical platform.

**Diagnosis:** the diagnosis of post-traumatic intralenticular abscess was established. There was no other diagnosis to discuss. The prognosis was relatively good since the infection was limited to the lens.

**Therapeutic interventions:** the patient was immediately started on an hourly instillation of topical fortified vancomycine and ceftazidime, topical corticosteroids and adjuvant treatment (cycloplegics and artificial tear drops). We also prescribed a large spectrum systemic antibiotic and antifungal association made of ofloxacine 200mg twice a day, imipenem 200mg twice a day and Voriconazole 200mg per day.

**Figure 4:**
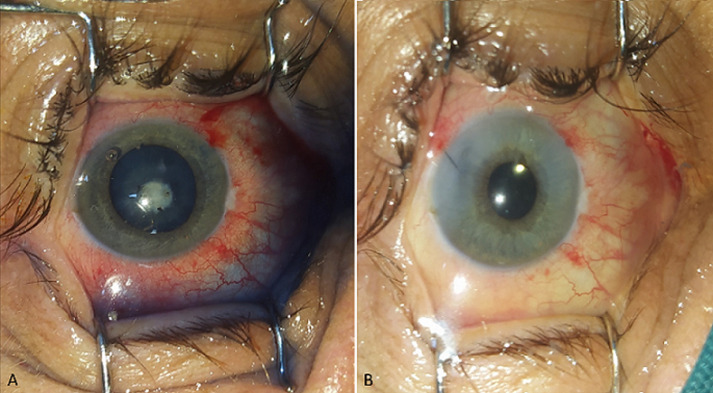
A) pre-operative photography of the right eye; B) post-operative photography of the right eye

**Figure 5:**
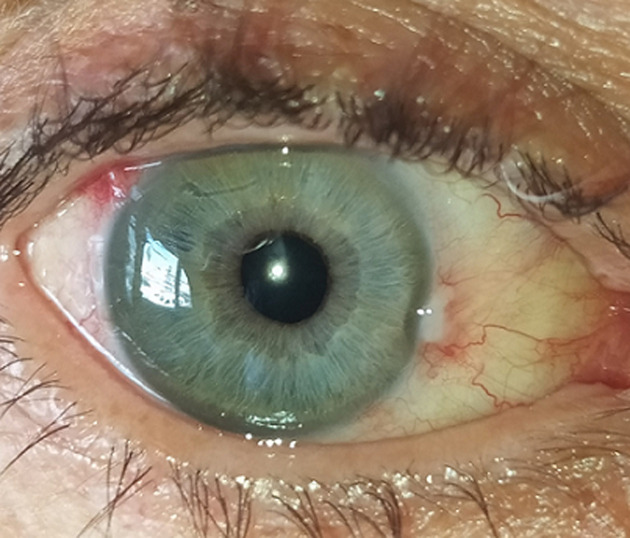
ocular aspect of the right eye at last follow-up

**Follow-up and outcome of interventions:** five days later, on this regimen, the condition improved, the anterior synechia was detached, the anterior chamber became quiet and the size and the depth of the lenticular abscess were unchanged (Figure 4A). On the seventh day, surgical management of the cataract was performed under topical anesthesia. A limbal incision was made using a 2.2mm keratome knife at 10 o'clock. Trypan blue was injected to aid visualization of the anterior capsule. An anterior continuous curvilinear capsulorhexis of approximately 5.5mm diameter was performed using a capsulorhexis forceps. The “drain of the abscess” was first attempted and immediately sent for microbiologic analysis. Afterwards, the aspiration of the whole lens was done using the irrigation/aspiration probe (Alcon Centurion® Vision system phacoemulsifier). A meticulous cortical cleanup was realized. In-the-bag intra ocular lens implantation of a single-piece hydrophobic acrylic intraocular lens (IOL) Acrysof SA60AT (Alcon Laboratories, USA) was attempted. The wound was sutured with 10-0 monofilament nylon. At the end of the surgery, an intracameral cefuroxime and a subconjunctival dexamethasone injection were performed (Figure 4B). Post operatively, antibiotics, antifungal drugs, and systemic steroid therapy were prescribed for four weeks. The culture of the lens was positive for staphylococcus epidermidis and the systemic treatment was adapted to the antibiogram. Slit lamp examination was daily performed and ultrasound B scan was made twice a week. The anterior and posterior segments remained quiet and final visual acuity at last follow-up, six months after surgery, was 9/10 (Figure 5).

**Patient perspective:** “I have always been a young man full of energy, the accident was such a hard experience for me. The first time I came to the emergency I was not able to see anything, I thought that my right eye was definitely damaged. Thanks to all the medical staff, I can read again and perform all my activities as usual. Hopefully this accident will only remain a bad memory”.

**Informed consent:** a written informed consent was obtained from the patient.

## Discussion

The primary complication of the intralentricular abscess is the chronic endophthalmitis [3]. In this report, we describe a case of intralenticular abscess complicating a thorn injury, successfully managed by surgical and medical approach. This rare condition results mainly from traumatic accidents. Shaped weapons such as needles or thorns are commonly causative agents. The main clinical feature is generally a white mass infiltrating the crystalline lens associated with a mild anterior chamber reaction and hypopyon [4]. In our patient, the lens abscess was evident with 3+ tyndall reaction without hypopyon but iris synechia. This condition could have been better documented using optical coherence tomography (OCT) of the anterior segment or ultrasound bioMicroscopy (UBM). The surgery was done after controlling the initial inflammation with systemic steroids associated to a wide spectrum intravenous, topical antibiotherapy and antifungal drugs. A phacoaspiration was performed under topical anesthesia. Since there was no vitreous reaction nor posterior capsule rupture, we haven´t performed posterior surgery nor intravitreal antibiotics injection as reported in previous works [5]. Microbiological proof of the infection can be obtained by different ways. Aqueous humor sampling may be negative while culture from of the crystalline cortex can reveal causative germs. Moreover, the culture of the entire lens seems to be the most effective way to ensure a contributive culture, since it is poorly penetrated by antibiotics [6].

Staphylococcus epidermidis is the most frequent implicated agent, but other microorganisms have been described in the literature such as pneumococcus, pseudomonas, propionibacterium acnes in addition to fungal agents. In some cases no causative agent has been individualized [7]. Staphylococcus epidermidis found in the sample of the lens aspirate was presumably inoculated directly from the thorn in the lens through the corneal wound. The early surgical management of the abscess was probably the main leading to a good visual outcome. Rajaraman *et al*.have shown the efficacity of early surgery to regain a good visual acuity [8]. Actually, as the lens is an avascular component, its penetration by antibiotics is difficult. Besides, the lens extraction allows a better efficiency of intracameral antibiotic injection [9]. What differs from one surgeon to another is the way of browsing the lens. In most cases authors propose a pars plana approach associated with a vitrectomy. Controverses remain concerning primary or secondary IOL implantation. “The one-step solution” based on cataract extraction and primary implantation allows an immediate visual rehabilitation. However, it would be associated to an increased risk of remaining infection and later ocular hypertension [10]. In our case, the primary IOL implantation was decided for three reasons: first, the inflammation of the anterior chamber was successfully controlled within five days from presentation. The second reason was the good corneal status [5]. The corneal perforation was sealed quickly, as thorns have a sharp margin and are relatively thin. Finally, the lack of vitreal involvement allowed an anterior segment surgery without posterior segment approach, thus simultaneous IOL implantation [4].

## Conclusion

Intralenticular abscess is a rare threatening-sight entity. The only effective way to preserve a good visual acuity is early lens extraction with adapted post-operative antibiotherapy. Regarding the timing of IOL implantation, the attitude depends on several features mainly the general ocular context and the preoperative care.
